# Postoperative Reduction Quality May Be the Most Important Factor That Causes Worse Functional Outcomes in Open and Closed Pelvic Fractures

**DOI:** 10.1007/s00268-021-06386-9

**Published:** 2022-01-01

**Authors:** Chih-Yang Lai, Po-Ju Lai, I-Chuan Tseng, Chun-Yi Su, Yung-Heng Hsu, Ying-Chao Chou, Yi-Hsun Yu

**Affiliations:** 1 Department of Orthopedic Surgery, Musculoskeletal Research Center, Chang Gung Memorial Hospital, Linkou Branch, and Chang Gung University, Tao-Yuan, Taiwan. 5, Fu-Hsin St. Kweishan, 33302 Tao-Yuan, Taiwan; 2 Department of Orthopedic Surgery, Chang Gung Memorial Hospital, Taoyuan Branch, Tao-Yuan City, Taiwan; 3 Department of Orthopedic Surgery, Chang Gung Memorial Hospital, Keelung Branch, Kee-Lung City, Taiwan

## Abstract

**Background:**

Data on the functional outcomes of patients with open pelvic fractures after osteosynthesis are limited, and whether open fracture is a risk factor for worse outcomes, as compared with closed fracture, remains unclear. This study aimed to compare the functional outcomes of patients with open and closed pelvic fractures and evaluate potential factors that might affect outcomes.

**Methods:**

Overall, 19 consecutive patients with open pelvic fractures and 78 patients with closed pelvic fractures between January 2014 and June 2018 were retrospectively reviewed. All fractures were surgically treated, with a minimal follow-up period of three years. Patients’ demographic profile, associated injuries, management protocol, quality of reduction, and outcomes were recorded and analyzed.

**Results:**

Patients with open pelvic fractures had higher new injury severity score, higher incidence of diverting colostomy, and longer length of stay. Both radiological and functional evaluations revealed no significant differences between the two groups at 1-year and 3-year evaluations. Multiple logistic regression analysis identified poor radiological outcomes (using Lefaivre criteria) and longer length of stay as risk factors for worse short-term functional outcomes. At 3-year evaluation, fair-to-poor radiological outcomes (using Matta/Tornetta and Lefaivre criteria) and the presence of diverting colostomy were potential risk factors.

**Conclusions:**

Compared with closed pelvic fracture, open pelvic fracture was not an indicator of worse functional outcomes. Functional outcomes may be comparable between patients with open and closed pelvic fractures at different time points within three years postoperatively. Achieving anatomical reduction in a fracture is crucial, because it might affect patient satisfaction.

## Introduction

Pelvic fractures account for 1%–3% of all skeletal injuries, and the management remains challenging for orthopedic surgeons [1]. Significant functional morbidities and a relatively high rate of mortality, ranging from 10 to 16%, may be hypothetically associated with pelvic fractures [2–4]. Therefore, optimal treatment methods to save lives and preserve a satisfactory functional status in patients, following pelvic fracture, are being developed.

Owing to improvements in prehospital care, diagnostic tools, resuscitation protocols, damage control procedures, evidence-based algorithms, and multidisciplinary teamwork, the morbidity and mortality rates in patients with pelvic fractures have decreased during the past decade [3, 5–7]. Meanwhile, improvements in the understanding of the pelvic anatomy and concepts of osteosynthesis have led to the expansion of indications for surgical intervention to maximize functional outcomes in these patients [8]. However, several studies have reported that various factors, such as fracture type, sex, age, injury severity score, associated injuries, surgical method, and quality of reduction, might affect post-injury functional outcomes [9–15].

Open pelvic fractures are considered more severe than closed fractures because of the presence of hemodynamic instability, high possibility of soft tissue damage and loss, and high risk of surgical site infection [3, 16, 17]. Functional deficits may theoretically be more significant in patients with open pelvic fractures. To date, only a few studies have addressed the functional outcomes of open pelvic fractures and explored the factors that may cause worse outcomes compared with those of closed fractures. Thus, this study aimed to directly compare functional outcomes after osteosynthesis between open and closed pelvic fractures and investigate the factors that might differentiate outcomes.

## Material and methods

We retrospectively reviewed the medical and radiological records of patients with pelvic fractures who underwent osteosynthesis between January 2014 and June 2018 at our institute from the institutional trauma registry. The review process was approved by the Institutional Review Board (IRB No. 202101095B0).

The inclusion criteria for this study were as follows: (1) patients with pelvic fracture who underwent osteosynthesis, (2) patients who received complete medical and radiological follow-up for at least 12 months, and (3) patients who received complete functional evaluations for more than 36 months. Patients who received conservative treatments, those who had concomitant acetabular fractures, those who were lost to follow-up, and those who did not receive complete functional evaluation were excluded from the study. Patients’ demographic profile, injury severity score, new injury severity score, fracture pattern-associated injuries, management protocol, quality of reduction, and outcomes were recorded.

### Resuscitation and osteosynthesis protocol

All patients were resuscitated based on Advanced Trauma Life Support guidelines upon arrival at the emergency department. Since transarterial embolization is a key procedure during resuscitation for pelvic fractures in our institute, it was performed when patients were not responding to blood transfusion and contrast agent pooling, as detected on computed tomography (CT), after the exclusion of other sources of hemorrhage.

Osteosynthesis for pelvic fractures was performed as early as possible when the patient’s clinical condition had been stabilized. The surgical approaches and choices of implants for definite treatment were primarily based on the classification of the fracture, distance of fracture displacement, and concomitant visceral or skeletal injuries. Generally, Pfannenstiel and ilioinguinal approaches are the major approaches for ventral ring injury when open reduction and internal fixation (ORIF) are indicated. In cases wherein fracture could be treated via closed reduction and internal fixation (CRIF), percutaneous fixation using screws was performed. External skeletal fixator for ventral ring injury was considered a bridging treatment (between the time of injury and the time of osteosynthesis) or a definite treatment in cases of inaccessible internal fixation from ventral visceral injuries.

For dorsal pelvic ring injury, which is a crescenteric fracture of the iliac wing, sacroiliac joint diastasis, and sacral fracture, CRIF with an iliosacral or trans-iliac-trans-sacral screw was the first choice for osteosynthesis. However, when anatomical reduction could not be achieved via the closed method or a global instability was present, ORIF using screws or plates or spinopelvic osteosynthesis was indicated and preferred.

### Fracture classification and radiological evaluations

Pelvic fractures were classified, based on the AO/OTA classifications system (2018 revision), to determine the stability of pelvic ring injury, which include the following three types: stable ring, partially stable ring, and unstable ring [18]. Two classification systems were used to identify open pelvic fractures: Faringer and Jones-Powell classifications, which focus on the location of the open wound and the location of the open wound with fracture stability, were specifically used to classify open pelvic fractures [19, 20].

Radiological outcomes were determined through postoperative plain radiographs using three standard pelvic radiographic views: anteroposterior, inlet, and outlet views. Regarding the quality of post-osteosynthesis, we adapted the Matta/Tornetta and the Lefaivre criteria to evaluate vertical displacement and pelvic symmetry, respectively (Table 1) [21–26].

**Table 1:** Grading of radiological outcomes based on Matta/Tornetta and Lefaivre criteria [21]

	Matta/Tornetta criteria Displacement (mm)	Lefaivre criteria Displacement (mm)
Excellent	<4	<5
Good	4–10	5–10
Fair	10–20	10–20
Poor	>20	>20

### Functional evaluations

Functional outcomes were assessed using Merle d’Aubigné score and Majeed score at 3, 6, 12, 24, and 36 months after injury in all patients. Merle d’Aubigné score includes parameters for pain, mobility, and walking ability, with each parameter rated from 0 points (worst condition) to 6 points (best condition); a high score represents good hip function [27, 28]. Majeed score is a pelvic injury-specific functional assessment that comprises seven items, including pain, work, sitting, sexual intercourse, standing, unaided gait, and walking distance, with a total score range of 0–100, in order of decreasing disability [13, 29]. Outcomes were graded as excellent (score >85), good (score of 70–84), fair (score of 55–69), and poor (score <55). For statistical analysis, patients were classified into two groups according to the Majeed score: satisfactory outcome, including excellent (score >85) and good (score of 70–84), and unsatisfactory outcome, including fair (score of 55–69) and poor (score <55).

### Statistical analysis

Data were analyzed using SPSS software version 23.0 (SPSS Inc., Chicago, IL, USA). Statistical significance was set at a *p* value of <0.05. Continuous variables were compared using Student’s *t* test, whereas categorical variables were compared using the chi-squared test and Fisher’s exact test. Multivariate logistic regression was used to determine potential risk factors for unsatisfactory functional outcomes.

## Results

During the 42-month study period, we identified a consecutive series of 97 patients, including 19 patients with open pelvic fractures and 78 patients with closed pelvic fractures who underwent osteosynthesis and met the inclusion criteria. ORIF using a plate was applied in 71 patients, while the other 26 patients underwent CRIF using a screw or an external fixator. For anterior ring injury, the Pfannenstiel approach was applied in 23 patients, and the ilioinguinal approach was applied in 22 patients. Regarding posterior ring injury, the approaches included CRIF with an iliosacral or trans-iliac-trans-sacral screw in 45 patients, ORIF for sacroiliac joint lesions in seven patients, triangular osteosynthesis in two patients, and spinopelvic osteosynthesis in 17 patients.

Patients with open pelvic fractures had a higher new injury severity score (27 vs. 17, *p* = 0.01), higher incidence of diverting colostomy (9 [47.4%] vs. 2 [2.6%], *p* = 0.01), and longer hospital stay (25.7 days vs. 18.5 days, *p* = 0.014) than those with closed fractures (Table 2). However, no significant differences were observed in the number of associated injuries and fracture severity.

**Table 2:** Demographic characteristics of 97 patients with pelvic fractures after surgical fixation

	Open pelvic fractures	Closed pelvic fractures	*p* value
Number	19	78	
Age (years)	37.5 (IQR: 28)	39.6 (IQR: 31)	0.632
Sex, n (%)			0.308
Men	10 (52.6%)	31 (39.7%)	
Women	9 (47.4%)	47 (60.3%)	
Associate organ injury, n (%)			
Brain	1 (5.3%)	12 (15.4%)	0.453
Chest	6 (31.6%)	29 (37.2%)	0.649
Abdomen	4 (21.1%)	18 (23.1%)	0.850
Urogenital	5 (26.3%)	13 (16.7%)	0.337
Extremities	12 (63.2%)	45 (57.7%)	0.664
ISS (median)	20 (IQR: 16–27)	17 (IQR: 8–26)	0.081
NISS (median)	27 (IQR: 22–38)	17 (IQR: 12–30)	0.010
OTA classification^a^			0.058
Stable ring	4 (21.1%)	4 (5.1%)	
Partially stable ring	6 (31.6%)	37 (47.4%)	
Unstable ring	9 (47.4%)	37 (47.4%)	
Osteosynthesis, n (%)			0.600
ORIF	13 (68.4%)	58 (74.4%)	
CRIF or ESF	6 (31.6%)	20 (25.6%)	
Colostomy, n (%)	9 (47.4%)	2 (2.6%)	0.010
Length of stay (days)	25.7 (IQR: 11)	18.5 (IQR: 12.5)	0.014
Follow up (months)	39.1 (IQR: 3.5)	40.6 (IQR: 9)	0.652

*IQR* interquartile range, *ISS* injury severity score, *NISS* new injury severity score, *ORIF* open reduction and internal fixation, *CRIF* closed reduction and internal fixation, *ESF* external skeletal fixation

^a^The classification of pelvic fracture was based on the AO/OTA classification (2018 revision)

Table 3 shows a comparison of radiological and functional outcomes between patients with open pelvic fractures and patients with closed fractures. Although the comparisons of radiological outcomes revealed a higher percentage of malreduction using Matta/Tornetta criteria and equal levels of malreduction using Lefaivre criteria in patients with open pelvic fractures, the comparisons failed to show statistical differences. Similar results were also presented in functional outcome evaluations at each time point.

**Table 3:** Radiological outcomes and functional outcomes of patients with open and closed pelvic fractures

	Open pelvic fractures	Closed pelvic fractures	*p* value
Radiological outcomes Matta/Tornetta criteria			0.425
Excellent	11 (57.9%)	31 (39.7%)	
Good	5 (26.3%)	26 (33.3%)	
Fair	3 (15.8%)	16 (20.5%)	
Poor	0	5 (6.4%)	
Lefaivre criteria			0.148
Excellent	8 (42.1%)	18 (23.1%)	
Good	1 (5.3%)	20 (25.6%)	
Fair	7 (36.8%)	24 (30.8%)	
Poor	3 (15.8%)	16 (20.5%)	
Functional outcomes Merle d’Aubigné score			
3 months	4.7 ((IQR: 5.5)	5.6 ((IQR: 4)	0.295
6 months	8.9 (IQR: 6.5)	9.9 (IQR: 6)	0.303
12 months	12.6 (IQR: 5)	13.8 (IQR: 4)	0.192
24 months	13.8 (IQR: 4)	15.2 (IQR: 3)	0.155
36 months	14.4 (IQR: 3.5)	15.5 (IQR: 4)	0.231
*Majeed score*			
3 months	32.9 (IQR: 17)	38.0 (IQR: 23)	0.179
6 months	52.9 (IQR: 30.5)	55.7 (IQR: 22.5)	0.538
12 months	70.6 (IQR: 19)	70.9 (IQR: 25)	0.931
24 months	75.0 (IQR: 17)	78.5 (IQR: 18)	0.332
36 months	76.5 (IQR: 22)	80.9 (IQR: 20)	0.239

*IQR* interquartile range

The associations of the selected factors with the functional outcome (Majeed score) at different time points are demonstrated in Table 4 (1-year evaluation) and Table 5 (3-year evaluation). Moreover, 54 (54.3%) patients were satisfied with the 1-year postoperative outcome; however, 43 (45.7%) patients were not (Table 4). The analysis revealed that dissatisfaction was not significantly correlated with the nature of pelvic fracture (open or closed). However, factors, such as injury severity score, new injury severity score, OTA classification, brain injury, chest injury, urogenital injury, application of arterial embolization, length of stay, and radiological outcome, according to the Matta/Tornetta and Lefaivre criteria, seemed to affect 1-year functional performance. Further, multiple logistic regression analysis identified that potential factors, which were related to dissatisfaction, were poor reduction quality (Lefaivre criteria) (odds ratio [OR] 3.55, 95% confidence interval [CI] 1.07–11.76) and length of stay (OR 1.09, 95% CI 1.04–1.16) (Table 6).

**Table 4:** Factors associated with short-term (1 year after injury) functional outcomes

	Satisfactory outcome Majeed score ≥ 70	Unsatisfactory outcome Majeed score <70	*p* value
Number	54 (54.3%)	43 (45.7%)	
Age (years)	36.5 (IQR: 25)	42.5 (IQR: 31)	0.088
Sex			
Men	20 (37.0%)	21 (48.8%)	0.243
Women	34 (63.0%)	22 (51.2%)	
Open fracture Closed fracture	10 (18.5%) 44 (81.5%)	9 (20.9%) 34 (79.1%)	0.766
ISS (median)	10 (IQR: 7–21)	24 (IQR: 17–34)	0.001
NISS (median)	14 (IQR: 9–25)	27 (IQR: 18–36)	0.001
Associated organ injury			
Brain	3 (5.6%)	10 (23.3%)	0.011
Chest	12 (22.2%)	23 (53.5%)	0.001
Abdomen	10 (18.5%)	12 (27.9%)	0.273
Urogenital	6 (11.1%)	12 (27.9%)	0.035
Extremities	30 (55.6%)	27 (62.8%)	0.472
OTA classification			
Stable ring	7 (13.0%)	1 (2.3%)	0.001
Partially stable ring	30 (55.6%)	13 (29.4%)	
Unstable ring	17 (31.5%)	29 (70.6%)	
Osteosynthesis			
ORIF	38 (70.4%)	33 (76.7%)	0.481
CRIF or ESF	16 (29.6%)	10 (23.3%)	
Radiological outcome Matta/Tornetta criteria			
Excellent	28 (51.9%)	14 (32.6%)	0.020
Good	18 (33.3%)	13 (30.2%)	
Fair	8 (14.8%)	11 (25.6%)	
Poor	0	5 (11.6%)	
Lefaivre criteria			
Excellent	17 (31.5%)	9 (20.9%)	0.048
Good	15 (27.8%)	6 (14.0%)	
Fair	16 (29.6%)	15 (34.9%)	
Poor	6 (11.1%)	13 (30.2%)	
Length of stay (days)	15.6 (IQR: 9.5)	25.4 (IQR: 18)	0.001
Colostomy	4 (7.4%)	7 (16.3%)	0.207

*IQR* interquartile range, *ISS* injury severity score, *NISS* new injury severity score, *ORIF* open reduction and internal fixation, *CRIF* closed reduction and internal fixation, *ESF* external skeletal fixation

**Table 5:** Results of factors associated with mid-term (3 years after injury) functional outcomes

	Satisfactory outcome Majeed score ≥ 70	Unsatisfactory outcome Majeed score <70	*p* value
Number	80 (82.5%)	17 (17.5%)	
Age (years)	38.3 (IQR: 30)	43.5 (IQR: 28)	0.261
Sex men Women	36 (45.0%) 44 (55.0%)	5 (29.4%) 12 (70.6%)	0.237
Open fracture Closed fracture	14 (17.5%) 66 (82.5%)	5 (29.4%) 12 (70.6%)	0.314
ISS (median)	17 (IQR: 9–25)	20 (IQR: 17–34)	0.027
NISS (median)	17 (IQR: 12–29)	29 (IQR: 17–36)	0.022
*Associated organ injury*			
Brain	9 (11.3%)	4 (23.5%)	0.237
Chest	26 (32.5%)	9 (52.9%)	0.111
Abdomen	16 (20.0%)	6 (35.3%)	0.205
Urogenital	12 (15.0%)	6 (35.3%)	0.080
Extremities	47 (58.8%)	10 (58.8%)	0.996
OTA classification			0.035
Stable ring	8 (10.0%)	0	
Partially stable ring	38 (47.5%)	5 (29.4%)	
Unstable ring	34 (42.5%)	12 (70.6%)	
Osteosynthesis			0.348
ORIF	57 (71.3%)	14 (82.4%)	
CRIF or ESF	23 (28.7%)	3 (17.6%)	
*Radiological outcome*			
Matta/Tornetta criteria			0.001
Excellent	38 (47.5%)	4 (23.5%)	
Good	27 (33.8%)	4 (23.5%)	
Fair	15 (18.8%)	4 (23.5%)	
Poor	0	5 (29.4%)	
Lefaivre criteria			0.002
Excellent	26 (32.5%)	0	
Good	19 (23.8%)	2 (11.8%)	
Fair	24 (30.0%)	7 (41.2%)	
Poor	11 (13.8%)	8 (47.1%)	
Length of stay (days)	17.3 (IQR: 12.5)	32.3 (IQR: 12)	0.001
Colostomy	6 (7.4%)	5 (29.4%)	0.021

*IQR* interquartile range, *ISS* injury severity score, *NISS* new injury severity score, *ORIF* open reduction and internal fixation, *CRIF* closed reduction and internal fixation, *ESF* external skeletal fixation

**Table 6:** Results of multiple logistic regression analysis for risk factors associated with short-term and mid-term unsatisfactory functional outcomes

Risk factors	Short-term functional outcome		
	OR	Estimated 95% CI	*p* value
Length of stay	1.09	1.04–1.16	0.001
Poor radiological outcome using			
Lefaivre criteria	3.55	1.07–11.76	0.038

Risk factors	Mid-term functional outcome		
	OR	Estimated 95% CI	*p* value
Diverting colostomy	7.69	1.56–41.67	0.013
Fair-to-poor radiological outcome by			
Matta/Tornetta criteria	3.79	1.10–13.10	0.035
Lefaivre criteria	9.23	1.71–49.75	0.010

*OR* odds ratio*, CI* confidence interval

Similar functional outcomes were noted at the 3-year evaluation, as shown in Table 5. In addition, 80 (82.5%) patients were satisfied with the outcome; however, 17 (17.5%) patients were not. Thus, whether the pelvic fracture was open or closed remained uncorrelated with unsatisfactory outcomes at this time of evaluation. Further, multiple logistic regression analysis revealed that the potential factors related to dissatisfaction were fair-to-poor reduction quality using Matta/Tornetta and Lefaivre criteria (OR 3.79, 95% CI 1.10–13.10 and OR 9.23, 95% CI 1.71–49.75, respectively) and experience with the diverting colostomy procedure (OR 7.69, 95% CI 1.56–41.67) (Table 6).

## Discussion

In this study, we found that patients with open pelvic fractures had comparable functional outcomes with those with closed fractures at the 1-year and 3-year evaluations. We identified poor radiological outcome (using Lefaivre criteria) and length of stay as potential factors for unsatisfactory 1-year functional outcomes. Regarding the 3-year functional evaluation, fair-to-poor radiological outcome (using Matta/Tornetta and Lefaivre criteria) and the presence of diverting colostomy were potential factors related to unsatisfactory functional outcome.

A higher injury severity score was a risk factor for poorer functional outcomes in previous studies [8, 9, 15, 30]. Brouwers et al. reported that a high injury severity score was a prognostic factor for decreased health-related quality of life [15]. Patients with open pelvic fractures may hypothetically have a higher rate of mortalities and complications than those with closed fractures, as commonly reported in previous studies [6, 7, 31]. Frane et al. reported a mortality rate of 14%, with an inclusive complication rate of 48.5% in patients with open pelvic fractures [32]. However, data regarding the functional status of survivors of open pelvic fractures are sparse. Although patients with an open fracture have more severe injuries than those with a closed fracture, we did not observe any difference in functional performance between these patients at the 1-year and 3-year evaluations. We postulated that the similar functional performance in both groups could be attributed to the application of adequate resuscitation, universal perioperative management, and patient-specific rehabilitation protocols.

Whether the quality of reduction for pelvic fracture is a key factor that may influence functional outcomes remains controversial [12, 33–36]. Kokubo et al. reported that a pelvic ring displacement of over 20 mm negatively influenced functional outcomes [10]. Nepola et al. stated that the degree of residual vertical displacement does not affect functional outcome [35]. The study results are consistent with those of the study by Kokubo et al. The statistical analysis revealed that the radiological outcomes of patients with both open and closed pelvic fractures had a significant effect on functional outcomes at the 1-year and 3-year follow-up. A worse postoperative function was correlated with a greater residual fracture gap and a bilaterally asymmetrical pelvis.

Another factor that might have affected functional performance in our study was the diverting colostomy procedure. Diverting colostomy is usually required in patients with concomitant colorectal injuries, especially those with open pelvic fractures [31, 37]. Studies have recommended that early diverting colostomy for open pelvic fractures, especially for Faringer zone I injury, is a crucial step in preventing surgical site infection, sepsis, and multiple organ dysfunction [6, 19, 37, 38]. Since diverting colostomy is a necessary procedure in patients with open pelvic fractures, there was no definite time for the colostomy closure. In our patients, diverting colostomy was usually performed while the patients could freely ambulate, usually 4–6 months after osteosynthesis. Although no significant relationship was observed between diverting colostomy and functional performance at the 1-year evaluation, diverting colostomy had a negative effect on functional outcome at the 1-year and 3-year evaluations. A possible reason was that patients who required diverting colostomy originally had more complex injuries than those who did not require this procedure. Therefore, the negative effect of pelvic fracture complexity was reflected by the diverting colostomy procedure.

Although we attempted to avoid bias, some limitations existed in our study. First, the retrospective design was inherently associated with the risk of recall bias, and the sample size was limited, because the data were obtained from a single institution. Second, some cases were excluded, given the lack of complete functional evaluations for more than 36 months. Third, this study had a short follow-up duration. Fourth, although we attempted to apply universally acceptable perioperative management for these patients, the resuscitative procedures might not have been completely similar among the patients, including the necessity of trans-arterial embolization, timing of diversional colostomy, and analysis of osteosynthesis. Finally, different procedures may produce different results. Nevertheless, further studies with larger sample size and a prospective design are required.

In conclusion, open pelvic fracture was not an indicator of worse functional outcomes. Contrarily, patients with open pelvic fractures might have comparable functional outcomes with those with closed fractures at different time points within three years. Length of stay and the diverting colostomy procedure had negative effects on functional performance in the 1-year and 3-year evaluations, respectively. Furthermore, poor fracture reduction was correlated with worse functional outcomes at the two evaluation time points.

## References

[1] Korovessis P, Baikousis A, Stamatakis M, Katonis P (2000). Medium- and long-term results of open reduction and internal fixation for unstable pelvic ring fractures. Orthopedics.

[2] Martínez F, Alegret N, Carol F, Laso MJ, Zancajo J, García E, Ros V (2018). Pelvic fracture in the patient with multiple injuries: factors and lesions associated with mortality. Emergencias.

[3] Mi M, Kanakaris NK, Wu X, Giannoudis PV (2020). Management and outcomes of open pelvic fractures: an update. Injury.

[4] Lai CY, Tseng IC, Su CY, Hsu YH, Chou YC, Chen HW, Yu YH (2020). High incidence of surgical site infection may be related to suboptimal case selection for non-selective arterial embolization during resuscitation of patients with pelvic fractures: a retrospective study. BMC Musculoskelet Disord.

[5] Ghosh S, Aggarwal S, Kumar P, Kumar V (2019). Functional outcomes in pelvic fractures and the factors affecting them- A short term, prospective observational study at a tertiary care hospital. J Clin Orthop Trauma.

[6] Tseng IC, Chen IJ, Chou YC, Hsu YH, Yu YH (2020). Predictors of acute mortality after open pelvic fracture: experience from 37 patients from a level I trauma center. World J Surg.

[7] Siada SS, Davis JW, Kaups KL, Dirks RC, Grannis KA (2017). Current outcomes of blunt open pelvic fractures: how modern advances in trauma care may decrease mortality. Trauma Surg Acute Care Open.

[8] Gabbe BJ, Hofstee DJ, Esser M, Bucknill A, Russ MK, Cameron PA, Handley C, de Steiger RN (2015). Functional and return to work outcomes following major trauma involving severe pelvic ring fracture. ANZ J Surg.

[9] Kabak S, Halici M, Tuncel M, Avsarogullari L, Baktir A, Basturk M (2003). Functional outcome of open reduction and internal fixation for completely unstable pelvic ring fractures (type C): a report of 40 cases. J Orthop Trauma.

[10] Kokubo Y, Oki H, Sugita D, Takeno K, Miyazaki T, Negoro K, Nakajima H (2017). Functional outcome of patients with unstable pelvic ring fracture. J Orthop Surg (Hong Kong).

[11] Sharpe JP, Magnotti LJ, Gobbell WC, Huang X, Perez EA, Fabian TC, Croce MA (2017). Impact of early operative pelvic fixation on long-term self-reported outcome following severe pelvic fracture. J Trauma Acute Care Surg.

[12] Suzuki T, Shindo M, Soma K, Minehara H, Nakamura K, Uchino M, Itoman M (2007). Long-term functional outcome after unstable pelvic ring fracture. J Trauma.

[13] Lefaivre KA, Slobogean GP, Valeriote J, O’Brien PJ, Macadam SA (2012). Reporting and interpretation of the functional outcomes after the surgical treatment of disruptions of the pelvic ring: a systematic review. J Bone Joint Surg Br.

[14] Dienstknecht T, Pfeifer R, Horst K, Sellei RM, Berner A, Zelle BA, Probst C, Pape HC (2013). The long-term clinical outcome after pelvic ring injuries. Bone Joint J.

[15] Brouwers L, de Jongh MAC, de Munter L, Edwards M, Lansink KWW (2020). Prognostic factors and quality of life after pelvic fractures. The brabant injury outcome surveillance (BIOS) study. PLoS ONE.

[16] Dente CJ, Feliciano DV, Rozycki GS, Wyrzykowski AD, Nicholas JM, Salomone JP, Ingram WL (2005). The outcome of open pelvic fractures in the modern era. Am J Surg.

[17] Westhoff J, Höll S, Kälicke T, Muhr G, Kutscha-Lissberg F (2004). Open pelvic fracture: treatment strategy and results for 12 patients. Unfallchirurg.

[18] Meinberg EG, Agel J, Roberts CS, Karam MD, Kellam JF (2018). Fracture and dislocation classification compendium-2018. J Orthop Trauma.

[19] Faringer PD, Mullins RJ, Feliciano PD, Duwelius PJ, Trunkey DD (1994). Selective fecal diversion in complex open pelvic fractures from blunt trauma. Arch Surg.

[20] Jones AL, Powell JN, Kellam JF, McCormack RG, Dust W, Wimmer P (1997). Open pelvic fractures: a multicenter retrospective analysis. Orthop Clin North Am.

[21] Mataliotakis GI, Giannoudis PV (2011). Radiological measurements for postoperative evaluation of quality of reduction of unstable pelvic ring fractures: advantages and limitations. Injury.

[22] Lefaivre KA, Blachut PA, Starr AJ, Slobogean GP, O’Brien PJ (2014). Radiographic displacement in pelvic ring disruption: reliability of 3 previously described measurement techniques. J Orthop Trauma.

[23] Yu YH, Liu CH, Hsu YH, Chou YC, Chen IJ, Wu CC (2021). Matta’s criteria may be useful for evaluating and predicting the reduction quality of simultaneous acetabular and ipsilateral pelvic ring fractures. BMC Musculoskelet Disord.

[24] Tornetta P, Matta JM (1996). Outcome of operatively treated unstable posterior pelvic ring disruptions. Clin Orthop Relat Res.

[25] Pastor T, Tiziani S, Kasper CD, Pape HC, Osterhoff G (2019). Quality of reduction correlates with clinical outcome in pelvic ring fractures. Injury.

[26] Lefaivre KA, Starr AJ, Barker BP, Overturf S, Reinert CM (2009). Early experience with reduction of displaced disruption of the pelvic ring using a pelvic reduction frame. J Bone Joint Surg Br.

[27] D’Aubigne RM, Postel M (1954). Functional results of hip arthroplasty with acrylic prosthesis. J Bone Joint Surg Am.

[28] d’Aubigné RM, Postel M (2009). The classic: functional results of hip arthroplasty with acrylic prosthesis. 1954. Clin Orthop Relat Res.

[29] Majeed SA (1989). Grading the outcome of pelvic fractures. J Bone Joint Surg Br.

[30] Harvey-Kelly KF, Kanakaris NK, Obakponovwe O, West RM, Giannoudis PV (2014). Quality of life and sexual function after traumatic pelvic fracture. J Orthop Trauma.

[31] Fu CY, Huang RY, Wang SY, Liao CH, Huang JF, Hsu YP, Lin CY, Kang SC (2018). Concomitant external and internal hemorrhage: challenges to managing patients with open pelvic fracture. Am J Emerg Med.

[32] Frane N, Iturriaga C, Bub C, Regala P, Katsigiorgis G, Linn M (2020). Risk factors for complications and in-hospital mortality: an analysis of 19,834 open pelvic ring fractures. J Clin Orthop Trauma.

[33] Lindahl J, Hirvensalo E (2005). Outcome of operatively treated type-C injuries of the pelvic ring. Acta Orthop.

[34] Pohlemann T, Bosch U, Gänsslen A, Tscherne H (1994). The Hannover experience in management of pelvic fractures. Clin Orthop Relat Res.

[35] Nepola JV, Trenhaile SW, Miranda MA, Butterfield SL, Fredericks DC, Riemer BL (1999). Vertical shear injuries: is there a relationship between residual displacement and functional outcome?. J Trauma.

[36] Dujardin FH, Hossenbaccus M, Duparc F, Biga N, Thomine JM (1998). Long-term functional prognosis of posterior injuries in high-energy pelvic disruption. J Orthop Trauma.

[37] Fitzgerald CA, Moore TJ, Morse BC, Subramanian A, Dente CJ, Patel DC, Reisman WM, Schenker ML, Gelbard RB (2017). The role of diverting colostomy in traumatic blunt open pelvic fractures. Am Surg.

[38] Lunsjo K, Abu-Zidan FM (2006). Does colostomy prevent infection in open blunt pelvic fractures? A systematic review. J Trauma.

