# Correction to: Postoperative Reduction Quality May Be the Most Important Factor That Causes Worse Functional Outcomes in Open and Closed Pelvic Fractures

**DOI:** 10.1007/s00268-022-06603-z

**Published:** 2022-06-28

**Authors:** Chih-Yang Lai, Po-Ju Lai, I-Chuan Tseng, Chun-Yi Su, Yung-Heng Hsu, Ying-Chao Chou, Yi-Hsun Yu

**Affiliations:** 1 Department of Orthopedic Surgery, Musculoskeletal Research Center, Chang Gung Memorial Hospital, Linkou Branch, and Chang Gung University, Tao-Yuan, Taiwan, 5, Fu-Hsin St. Kweishan, 33302 Tao-Yuan, Taiwan; 2 Department of Orthopedic Surgery, Chang Gung Memorial Hospital, Taoyuan Branch, Tao-Yuan City, Taiwan; 3 Department of Orthopedic Surgery, Chang Gung Memorial Hospital, Keelung Branch, Kee-Lung City, Taiwan

## Correction to: World J Surg (2022) 46:568–576 10.1007/s00268-021-06386-9

In the original online version of this article the percentage in the second sentence on page 572 was incorrect. The correct value is 45.7%.

The original article was corrected.

